# Ultraviolet screening by slug tissue and tight packing of plastids protect photosynthetic sea slugs from photoinhibition

**DOI:** 10.1007/s11120-021-00883-7

**Published:** 2021-11-26

**Authors:** Vesa Havurinne, Riina Aitokari, Heta Mattila, Ville Käpylä, Esa Tyystjärvi

**Affiliations:** Department of Life Technologies/Molecular Plant Biology, University of Turku, Turku, Finland

**Keywords:** Action spectroscopy, Kleptoplasty, Photoinhibition, Photosynthetic sea slugs, Photosystem II, UV radiation

## Abstract

One of the main mysteries regarding photosynthetic sea slugs is how the slug plastids handle photoinhibition, the constant light-induced damage to Photosystem II of photosynthesis. Recovery from photoinhibition involves proteins encoded by both the nuclear and plastid genomes, and slugs with plastids isolated from the algal nucleus are therefore expected to be incapable of constantly repairing the damage as the plastids inside the slugs grow old. We studied photoinhibition-related properties of the sea slug *Elysia timida* that ingests its plastids from the green alga *Acetabularia acetabulum*. Spectral analysis of both the slugs and the algae revealed that there are two ways the slugs use to avoid major photoinhibition of their plastids. Firstly, highly photoinhibitory UV radiation is screened by the slug tissue or mucus before it reaches the plastids. Secondly, the slugs pack the plastids tightly in their thick bodies, and therefore plastids in the outer layers protect the inner ones from photoinhibition. Both properties are expected to greatly improve the longevity of the plastids inside the slugs, as the plastids do not need to repair excessive amounts of damage.

**Supplementary Information:**

The online version contains supplementary material available at 10.1007/s11120-021-00883-7.

## Introduction

*Elysia timida* belongs to a group of animals that carry out photosynthesis using plastids ingested from their prey. This interesting phenomenon, called kleptoplasty, has only been reported in Sacoglossan sea slugs like *E. timida* (Rumpho et al. 2011; de Vries et al. 2014) and marine flatworms (Van Steenkiste et al. 2019). The record holding photosynthetic slug *Elysia chlorotica* maintains kleptoplasts functional for approximately a year (Green et al. 2000), and has served as one of the most important subjects for the study of kleptoplastic animals (Chan et al. 2018; Cai et al. 2019). However, the limited availability of *E. chlorotica* individuals is a major obstacle for in-depth laboratory studies. *E. timida* is known for its easy husbandry in the laboratory (Schmitt et al. 2014; Havurinne and Tyystjärvi 2020). Use of laboratory cultures reduces stress on natural populations of sea slugs and offers controlled conditions that improve the reproducibility of the experiments. Plastids of the slug *E. timida* originate from the green alga *Acetabularia acetabulum* (hereafter *Acetabularia*).

Many questions related to photosynthetic sea slugs have no answer so far. For example, it is unclear how the slugs recognize and incorporate foreign organelles into their own cells. The uptake process has been suggested to involve the slug's innate immune system that can possibly recognize the plastids via scavenger receptors and thrombospondin‐type‐1 repeat proteins (Chan et al. 2018; Clavijo et al. 2020). It is also uncertain just how important are the native properties of the plastids themselves in terms of facilitating their survival for weeks and months inside animal cytosol in isolation from the algal nucleus. The slugs are only able to retain plastids that come from specific algae species (Christa et al. 2013; de Vries et al. 2013), but to what extent this is due to the general robustness of plastids of these algae (Giles and Sarafis 1972; Trench et al. 1973a; 1973b; Green et al. 2005) or their specific genetic and photosynthetic properties (de Vries et al. 2013; Christa et al. 2018; Havurinne et al. 2021) remains to be fully tested.

Another heavily debated issue is the physiological significance of functional plastid retention for the slugs over extended periods of time. When food is abundant, the capability to maintain the plastids functional might not be important for the slugs, as it was recently shown that the slug *Elysia viridis* continuously and actively digests old plastids and replaces them with new ones during feeding (Frankenbach et al. 2021). This would imply that the main nutritional value gained by the slugs from the algae during feeding comes simply from active digestion of the algal components, not from photosynthesis. However, long-time survival of continuously fed *Elysia atroviridis* slugs was better under moderate than weak light, which supports a different view (Shiroyama et al. 2020). Some of the photosynthetic sea slugs can experience seasonal food scarcity, like the Mediterranean species *E. timida* during the autumn and spring when its prey alga *A. acetabulum* is mainly found in its calcified form that is impenetrable for the slug (Marín and Ros 1992). It is generally accepted that in such times the slugs do benefit from the plastids, but whether this benefit is achieved by active transport of photosynthates or simply slow digestion of the plastids remains controversial (Christa et al. 2014; Cartaxana et al. 2017; Laetz et al. 2017; Frankenbach et al. 2021). It was recently suggested that metabolites produced in the plastids could be directed specifically for reproduction rather than adult slug household metabolism (Cruz et al. 2020; Cartaxana et al. 2021).

Irreversible light-induced damage to Photosystem II (PSII) of the photosynthetic electron transfer chain, termed photoinhibition, is an important reason why survival of plastids in isolation within slug cells for months requires special mechanisms. Photoinhibition, the paradoxical downside of utilizing light energy to run photosynthesis, has been shown to be ubiquitous in photosynthetic organisms, and it occurs even in low light (Tyystjärvi and Aro 1996). Even though the exact mechanism(s) of photoinhibition remain elusive, decades of work on the topic have revealed several “rules” that most photosynthetic organisms comply to. These rules include the following: (I) direct proportionality of the rate constant of the damaging reaction with photosynthetic photon flux density (PPFD), (II) the damaging reaction proceeds according to first-order reaction kinetics, and (III) UV radiation is considerably more damaging than visible light (Tyystjärvi 2013). In spite of photoinhibition, photosynthetic organisms maintain high photosynthetic activity by continuously repairing damaged PSII reaction centers (Järvi et al. 2015). Because the repair cycle of PSII is efficient, the actual rate of photodamage to PSII can be measured only if the repair cycle is blocked with an antibiotic that blocks plastid translation, such as lincomycin.

Even though resilience against the damaging reaction of photoinhibition might explain the longevity of plastids inside photosynthetic sea slugs, very few studies have addressed this directly. The effect of different intensities of light on the longevity of PSII activity in the plastids of *E. timida* and *E. viridis* has been evaluated, and the results show that stronger light during starvation leads to shorter retention of functional plastids (Vieira et al. 2009; Christa et al. 2018). On the other hand, much effort has been invested into evaluating the physiological photoprotection mechanisms of the plastids. It has been shown that the plastids in the slugs maintain similar or slightly elevated photoprotective non-photochemical quenching (NPQ) mechanisms as the plastids in the algae, at least in recently fed slugs (Cruz et al. 2015; Christa et al. 2018; Cartaxana et al. 2019; Havurinne and Tyystjärvi 2020). However, the effectiveness of these NPQ mechanisms in preventing net photoinhibition in *E. timida* in the absence of lincomycin remains controversial (Christa et al. 2018; Cartaxana et al. 2019). Photoinhibition and subsequent recovery of PSII in photosynthetic sea slugs in the presence of lincomycin has only been evaluated twice; once by Christa et al. (2018) in *E. timida* and *E. viridis,* and recently by us in *E. timida* (Havurinne et al. 2021). The results of these two studies contradict each other, as Christa et al. did not find any significant differences in the recovery of PSII in the presence or absence of lincomycin, whereas our data showed that lincomycin efficiently blocks the recovery process in photoinhibited plastids of *E. timida*. Our results would imply that freshly fed slugs do retain some capacity for PSII repair even without the help of the algal nucleus that encodes many of the proteins that are known to be related to the repair cycle at least in plants (Järvi et al. 2015; Havurinne et al. 2021). However, additional support for the existence of deficiencies in the PSII repair cycle in the slug plastids is available in the literature. Vieira et al. (2009) showed that PSII functionality and related parameters of the plastids in *E. viridis* individuals deprived of their food (starved) decrease according to first-order reaction kinetics throughout the starvation period even without lincomycin, especially under moderately bright light (PPFD 140 µmol m^−2^ s^−1^), suggesting that the repair cycle is not able to keep up with photoinhibition. The PSII activity in *E. timida* individuals starved in similar light conditions (250 µmol m^−2^ s^−1^) also exhibits first-order decay kinetics (Christa et al. 2018). These points clearly emphasize the need for further studies into both the actual damaging reactions and recovery processes from photoinhibition in photosynthetic sea slugs.

To our knowledge, all reports on plastid longevity in the slugs pertain to lab experiments with light sources that do not emit UV radiation, or to conditions where the natural sunlight is filtered through an UV radiation obstructing panel like glass. Photosynthetic sea slugs generally inhabit shallow coastal waters, and *E. timida* can often be found at 0.5–2 m depth (personal observation by Vesa Havurinne) where they can be exposed to high levels of solar irradiation (Giménez–Casalduero et al. 2011). However, it is unclear if the plastids are damaged by UV radiation inside the slugs. Here, we set out to thoroughly examine the characteristics of the damaging reactions of photoinhibition in the sea slug *E. timida* and its prey green alga *Acetabularia*, with the idea of testing which of the rules of photoinhibition hold true in the slugs. While our photoinhibition experiments show that the slugs are governed by the same basic principles of photoinhibition as their algal counterparts, properties of the slug tissue and placement of the plastids inside slug cells drastically slow down photoinhibition of the plastids inside the slug *E. timida*.

## Materials and methods

### Organisms and culture conditions

The sea slug *E. timida* (strain TI1) and its prey green alga *Acetabularia* (strain DI1; originally isolated by Diedrik Menzel) were maintained in 10 l plastic tanks at 23 °C in a 12/12 h day/night cycle (PPFD 40–50 µmol m^−2^ s^−1^ during the day), as described earlier (Havurinne and Tyystjärvi 2020). *E. timida* was cultured in 3.7% (m/v) artificial sea water (ASW; Sea Salt Classic; Tropic Marin, Montague, MA, USA). *Acetabularia* was cultured in f/2 medium made into 3.7% ASW. The slug tanks were continuously aerated. Experiments with *E. timida* were mainly done with freshly fed individuals, but slugs that had been kept in starvation (removed from the algal food source) for different time periods in their growth conditions were used in certain experiments, as indicated in the text. For the starvation treatments, the slugs were deprived of their food, and kept in 5 l tanks filled with ASW. The starving slugs were moved to clean tanks with fresh ASW weekly.

### Photoinhibition treatments

*Elysia timida* individuals of similar size and green color were selected for the photoinhibition treatments and subjected to overnight darkness in ASW containing 10 mg/ml lincomycin, a translation inhibitor shown to be plastid specific in plants (Mulo et al. 2003). *Acetabularia* samples selected for photoinhibition treatments were treated in an identical manner to the slugs in f/2 medium in the absence or presence of 10 mg/ml lincomycin. Lincomycin concentrations that have been used to block the PSII repair cycle in the plastids of photosynthetic sea slugs are high, for example 8 mg/ml (Christa et al. 2018) or 10 mg/ml (Havurinne et al. 2021). We opted to use the same concentration of lincomycin for the slugs and the algae because preliminary experiments showed that even in the algae a high lincomycin concentration is required to block PSII recovery after photoinhibition (Fig. S1 in Online Resource). Effectiveness of 10 mg/ml lincomycin in stopping PSII repair cycle in the slug plastids has been illustrated previously (Havurinne et al. 2021).

After an overnight incubation in the dark with lincomycin, slugs, or algae were placed inside the wells of a 24-well plate in their respective medium. For most of the experiments one slug individual or 3–5 strands of algae were placed inside a single well of the well plate, representing one biological replicate. The well plate bottom for both species was covered with aluminum foil to ensure that the slugs receive as much light as possible, as they tend to curl up next to the edges of the wells when exposed to high light. The wells were large enough to prevent the algal strands from shading each other in the same well. The ratio of variable to maximum chlorophyll (Chl) *a* fluorescence (*F*_V_/*F*_*M*_) was measured with a pulse amplitude modulation fluorometer PAM-2000 (Walz, Effeltrich, Germany), and used as a proxy of PSII activity as described earlier (Havurinne and Tyystjärvi 2020). The *F*_V_/*F*_M_ parameter is known to function well as a probe of photoinhibitory damage (Tyystjärvi 2013) although it was recently shown that *F*_V_/*F*_M_ does not represent the maximum quantum yield of PSII (Sipka et al. 2021). The first *F*_V_/*F*_M_ value was measured from samples that had been dark acclimated overnight, and 20 min dark incubation was applied before *F*_V_/*F*_M_ measurements during the photoinhibition treatments. The samples were returned to light treatment thereafter. The rate constant of photoinhibition (k_PI_) was determined by fitting the decrease in *F*_V_/*F*_M_ to first-order decay kinetics (Tyystjärvi and Aro 1996) using SigmaPlot v.14.0 (Systat Software, Inc., San Jose, CA, USA); time was measured as the cumulative illumination time, excluding the 20-min dark incubations.

White light for the photoinhibition treatments was provided by an Artificial Sunlight Module (SLHolland, Breda, The Netherlands; see Fig. S2 in Online Resource for the irradiance spectrum). The action spectra of photoinhibition were measured by exposing the samples to monochromatic light of different wavelengths. Although the emission spectra of the light sources were wide in some cases, the visible spectrum light treatments will be referred to as 690, 660, 560, 470, and 425 nm and those of the UV spectrum as 365 (UVA), 312 (UVB), and 254 nm (UVC). Monochromatic visible light used in the photoinhibition experiments was obtained using a custom-built LED array equipped with one of the Andover Corporation line filters 690FS, 660FS, 560FS, and 470FS (Newport, Irvine, CA, USA), where numbers stand for the respective center wavelengths of the filters; the full width at half maximum of these filters is 10 nm. 425 nm light was obtained using the Artificial Sunlight Module (SLHolland) in combination with 450 nm short pass and 400 nm long pass filters (Newport Corporation, Franklin, MA, USA), and the UV sources were VL-8.LC (UVA and UVC) and VL-8.M (UVB) UV lamps (Vilber, Marne la Vallée, France). PPFDs (or photon flux density, PFD, for UV radiation) of the photoinhibition treatments were measured from the water surface levels of the 24-well plates initially either with a planar, wavelength calibrated light sensor (LI-COR Biosciences) or with a planar STS-UV/visible light spectrometer (Ocean Optics, Largo, FL, USA). The aluminum foil placed underneath the well plates enhanced the PPFD of visible light (white light; Artificial Sunlight Module), and therefore PPFD values were determined also with a spherical underwater sensor (LI-COR Biosciences) from the wells filled with distilled water. PPFD measured with a planar sensor headed toward the light source showed only 1/3 of the PPFD measured with the spherical sensor because a spherical sensor accounts light from all directions, including reflection from the foil. The PPFDs of the visible light wavelengths 690, 660, 560, 470, and 425 nm were 900, 927, 402, 699, and 750 µmol m^−2^ s^−1^. The plastic of the well plate blocks UV radiation, and therefore the incident UV radiation of the samples was not enhanced to the same extent. UV radiation was measured only via the planar spectrometer probe, and the UVA, UVB, and UVC treatments were done with the PFDs of 33, 51 and 23 µmol m^−2^ s^−1^, respectively. After we had confirmed that k_PI_ is directly proportional to light intensity in *E. timida* and *Acetabularia*, we normalized the original k_PI_ values to P(P)FD 300 µmol m^−2^ s^−1^, but care should be taken when comparing the visible light action spectrum to the UV spectrum due to the differences in measurement of light. Furthermore, shading by slugs and algae reduces reflection from the foil to some extent.

### Room temperature fluorescence emission spectra

For comparison of Chl *a* fluorescence under excitation with different wavelengths, dark acclimated individual slugs or pieces of dark acclimated *Acetabularia* cells were placed on a dry, matte black cardboard and illuminated with low intensity monochromatic light to excite Chl *a*. The algae were cut with a razor blade, and the pieces, amounting to a similar sized clump as an individual slug, were placed in such a manner that the area they covered was similar to that of the slugs. Monochromatic light (450, 470, 490, 510, 530, 550, 590, 600, and 610 nm) was obtained from KL-1500 halogen light source (Schott AG, Mainz, Germany) filtered through Corion bandpass filters (full width at half maximum 10 nm) via fiber optic light guides. Fluorescence emission excited by 470 nm light was used as a control; the 470 nm excitation light was pointed at the samples at a 45° angle, with the head of the light guide fixed to approximately 5 mm away from the sample. Another end of the bifurcated light guide led the emitted fluorescence to the detector of the QE Pro spectrometer (Ocean optics). The light guide used for all other visible light wavelengths was also positioned at 45° toward the sample, opposite to the 470 nm light guide.

UV radiation was obtained from a UVA LED (Build My LED; https://www.buildmyled.com/) combined with a 390 nm Corion bandpass filter (full width at half maximum 10 nm). The UV-LED was placed perpendicular to the sample surface and 3 cm away from the sample, so that the end of the spectrometer’s light guide did not obstruct UV radiation. The PFD of the 390 nm excitation was 3 µmol m^−2^ s^−1^, measured with the STS-UV/visible light spectrometer (Ocean Optics), whereas the PPFDs of all other wavelengths were 4 µmol m^−2^ s^−1^, measured with a wavelength calibrated PPFD sensor (LI-COR).

For an individual *E. timida* or *Acetabularia* sample, the visible light measurements were carried out by first exciting the sample with 470 nm light and then switching excitation wavelengths (450–610 nm) while maintaining the sample at the exact same position. However, because the UV-excited fluorescence from the slugs was very weak and the correct placement of the samples had to be ensured, the UVA excited fluorescence was always measured first and then the 470 nm excited fluorescence. Fluorescence emission intensities obtained by using different excitation wavelengths were normalized, separately for each individual sample, to fluorescence emission at 685 nm region excited by 470 nm. The normalized fluorescence spectra from all biological replicates were then averaged.

### Confocal microscopy

Individual slugs and *Acetabularia* cells were imaged with an LSM880 confocal with an Axio Observer.Z1 microscope (Zeiss, Oberkochen, Germany) at the Cell Imaging and Cytometry Core, Turku Bioscience Centre, Turku, Finland, with the support of Biocenter Finland. The objective was 20 × Zeiss Plan-Apochromat and the acquisition software was ZEN 2.3 SP1. The samples were fixed overnight in the dark at 4 °C with 4% paraformaldehyde in PBS buffer containing 0.2% Tween-20. The slugs and the algae were placed in a welled microscope slide. The well was large enough to hold one slug, but the placement of a cover glass over the sample flattened the slug so that the parapodia stayed open. The algae were cut to small pieces to fit in the well. Chl fluorescence was excited with 633 nm light from a HeNe laser and emission was recorded with a GaAsp detector at 640–750 nm range. All images of the slugs were taken from the parapodia, one of the thinnest sections of the slug body, whereas with *Acetabularia* cells, pieces of the stalk were imaged. For Z-stacks, the sample was imaged at 3.8 µm intervals by setting the 0 µm layer at a level where Chl fluorescence was still clearly emitted, but nearly out of the focal range. Image analysis was performed with Fiji (Schindelin et al. 2012). Maximum intensity fluorescence projections were created using the Z-projection tool, where all slices of the Z-stack contributed to the projection. Average Chl fluorescence of each slice of the Z-stack was obtained by utilizing the “plot Z-axis profile” tool on the entire area of the images without selecting any specific regions of interest. The validity of this method in *Acetabularia*, where the cell area can be accurately defined, was confirmed by comparing the Chl fluorescence of each slice in Z-axis profiles without specific regions of interest to the profiles of Z-stacks where only the *Acetabularia* cell was selected as the region of interest.

### Absorptance and reflectance

Absorptance of intact *Acetabularia* cells and slugs was measured using an integrating sphere (Labsphere, North Sutton, NH, USA). The samples were placed inside a glass test tube in their respective media, and the tube was placed in the integrating sphere. Measurements of the empty sphere were performed with the tubes filled with the media. A 1000 W high pressure Xenon illuminator was used as a light source (Sciencetech Inc., London, Canada) and absorptance was measured with an STS-VIS spectrometer (Ocean Optics). The signal-to-noise ratio from the slugs was poor, and this was counteracted by placing 30 live slug individuals in the tube for the measurement. The signal from *Acetabularia* was clear, and approximately 5–10 *Acetabularia* cells were enough to return a sufficient signal for further analysis. All measurements were corrected with the absorptance measurements from absolutely calibrated matte black cardboard (Idle and Proctor 1983; Pätsikkä et al. 1998). However, absolute absorptance values were not calculated because the surface areas of the slugs and *Acetabularia* were unknown. Chls were extracted from the samples by overnight incubation in *N*,*N*-dimethylformamide (DMF) in the dark at 4 °C, and the total amounts of Chls were quantified spectrophotometrically using the wavelengths and extinction coefficients for Chls *a* and *b* in DMF (Porra et al. 1989).

A nearly identical experimental setup was used for the reflectance measurements as the one used for room temperature fluorescence. Individual slugs or multiple pieces of algae were placed on a matte black cardboard and illuminated with white light from a slide projector guided on to the sample with the bifurcated light guide of the QE Pro spectrometer (Ocean Optics). The distance between the probe and the sample was approximately 5 mm. For calibration, a white reflectance standard (Labsphere Inc.) was used to obtain full reflectance using the same setup. In addition to green slugs and *Acetabularia*, spectral reflectance was also measured from starved slug individuals that had lost some of their plastids during starvation.

## Results

### Photoinhibition is slower in E. timida than in Acetabularia

**Fig. 1:**
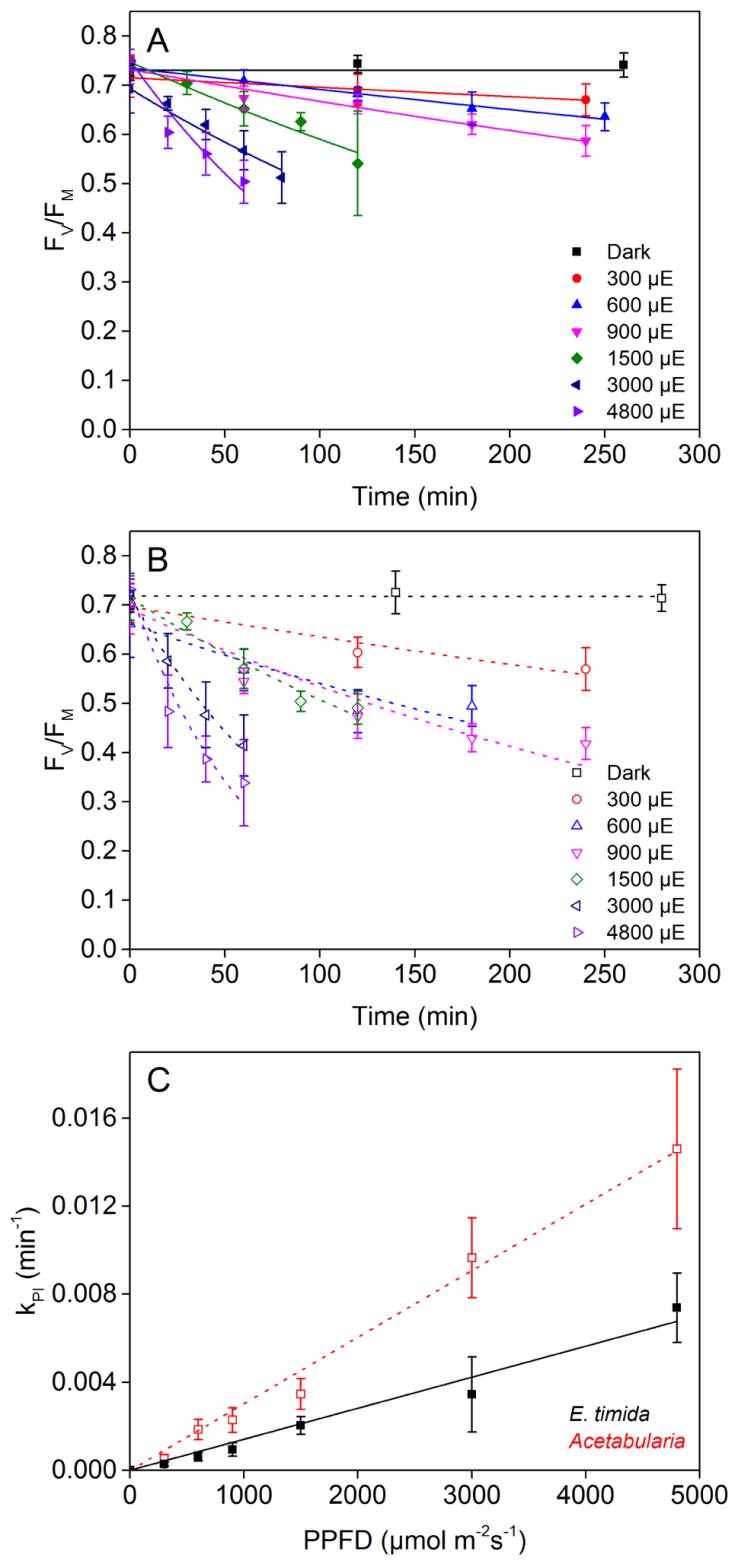
Light response of photoinhibition in lincomycin treated *Acetabularia* and *E. timida*. The decay of the fluorescence parameter *F*_*V*_/*F*_M_, a proxy of PSII activity, in **A** *E. timida* and **B** *Acetabularia* in response to different PPFDs, as indicated; the designation “µE” stands for µmol of photosynthetically active photons per square meter in a second. The lines show the best fits of the averaged data to first-order reaction kinetics (*R*^2^ of the fits ranged from 0.86 to 0.99 in *Acetabularia* and 0.93 to > 0.99 in *E. timida*). **C** Rate constants of photoinhibition (k_PI_) in *E. timida* (black) and *Acetabularia* (red) as a function of PPFD. The lines show linear regression (*R*^2^ = 0.99 and 0.98 for *Acetabularia* and *E. timida*, respectively). k_PI_ values were derived from the measurements shown in (**A**) and (**B**). Each data point represents an average of at least four biological replicates and the error bars show SD

We measured the decrease in the ratio of variable to maximum fluorescence (*F*_V_/*F*_M_) from both freshly fed *E. timida* and *Acetabularia* in the presence of lincomycin at seven different light intensities. The decrease in *F*_V_/*F*_M_ in both the slugs and the algae followed first-order reaction kinetics (Fig. 1A, B), as usual for photoinhibition of PSII (Tyystjärvi 2013). In both species the rate constants of photoinhibition (k_PI_) were directly proportional to light intensity, which indicates that photosynthetic sea slugs are not exempt from this core property of photoinhibition of PSII (Tyystjärvi and Aro 1996). The k_PI_ value, derived from the measurements in Fig. 1A, B, was approximately twice as high in the algae compared to the slugs in the tested PPFD range (Fig. 1C), suggesting that plastids inside *E. timida* are much less prone to photoinhibition of PSII than the plastids inside *Acetabularia* in our experimental conditions.

### Slug tissue protects the plastids by screening UV radiation

**Fig. 2:**
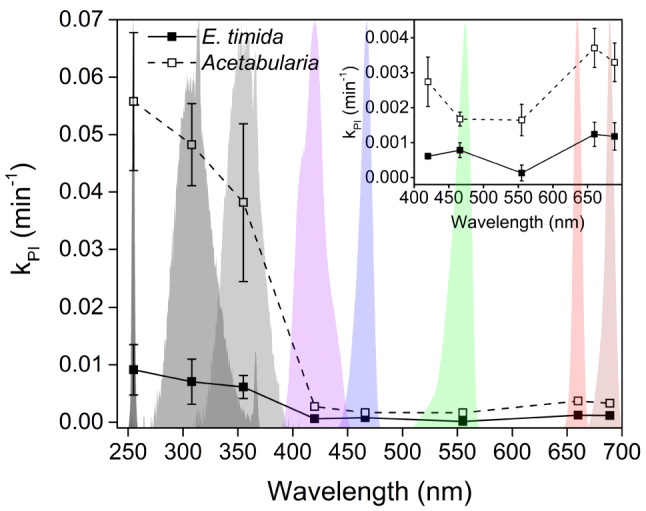
Action spectra of photoinhibition of *Acetabularia* and *E. timida*. The respective treatment light spectra are shown in the background. The rate constants of photoinhibition (k_PI_) have been normalized to (P)PFD (300 µmol m^−2^ s^−1^), the actual treatment light (P)PFDs are detailed in Materials and Methods. The inset shows a close up from the visible light wavelength range. Each k_PI_ was determined as the best fit to first-order reaction kinetics of the decrease in the fluorescence parameter *F*_V_/*F*_M_ during the photoinhibition treatments. Each data point represents an average of at least three biological replicates, and the error bars indicate SD

Next, we measured the action spectrum of photoinhibition from *Acetabularia* and *E. timida,* covering both UV and visible light regions. Since the UV radiation during the photoinhibition treatments was not enhanced by the aluminum foil placed on the bottom of the well plate used for illumination treatments to the same extent as the visible light (see Materials and Methods for details), absolute comparison of the k_PI_ values of the UV and visible light action spectra within one species should be done with caution. Nevertheless, UV radiation was found to be highly damaging to PSII in both organisms, especially *Acetabularia* (Fig. 2). In the visible light regions of the action spectra, both organisms showed distinct peaks of photoinhibition (Fig. 2 inset). These are common characteristics of photoinhibition shared by all photosynthetic organisms (Jones and Kok 1966; Havurinne and Tyystjärvi 2017; Soitamo et al. 2017, see Zavafer et al. 2015 for review). In both *Acetabularia* and *E. timida* the most pronounced peak in the visible light action spectra was in the red-light region, at 660 nm, but interestingly photoinhibitory efficiency did not significantly drop from 660 to 690 nm. However, the emission spectrum of the 690 nm light shows that our 690 nm source has a contribution of shorter wavelength light that may affect the results (Fig. 2). In *E. timida*, green 560 nm light caused very little photoinhibition, whereas a clear increase in photoinhibition from green to blue 460 and 420 nm light was noticeable. In *Acetabularia,* green 560 and blue 460 nm wavelengths were very similar in their damaging potential (Fig. 2 inset).

In the UV region photoinhibition increased as the wavelength shortened, with UVC causing the most rapid damage in both species. The k_PI_ values of *Acetabularia* and *E. timida* indicate that UV radiation inflicts very little photoinhibition in the slugs compared to the algae (Fig. 2). A slower rate of photoinhibition was already seen in white-light treatments of the slugs (Fig. 1), but the question remains, why does photoinhibition not increase with decreasing UV wavelength to the same extent in the slugs as in *Acetabularia*? Some of this effect might be explained by the fact that the UV radiation was not reflected by the aluminum foil to the same extent as visible light, making it possible that the slugs were able to protect themselves by curling up next to the well edges. However, we decided to next inspect the possibility of UV screening by the slug tissue as an extra protective measure against UV.

**Fig. 3:**
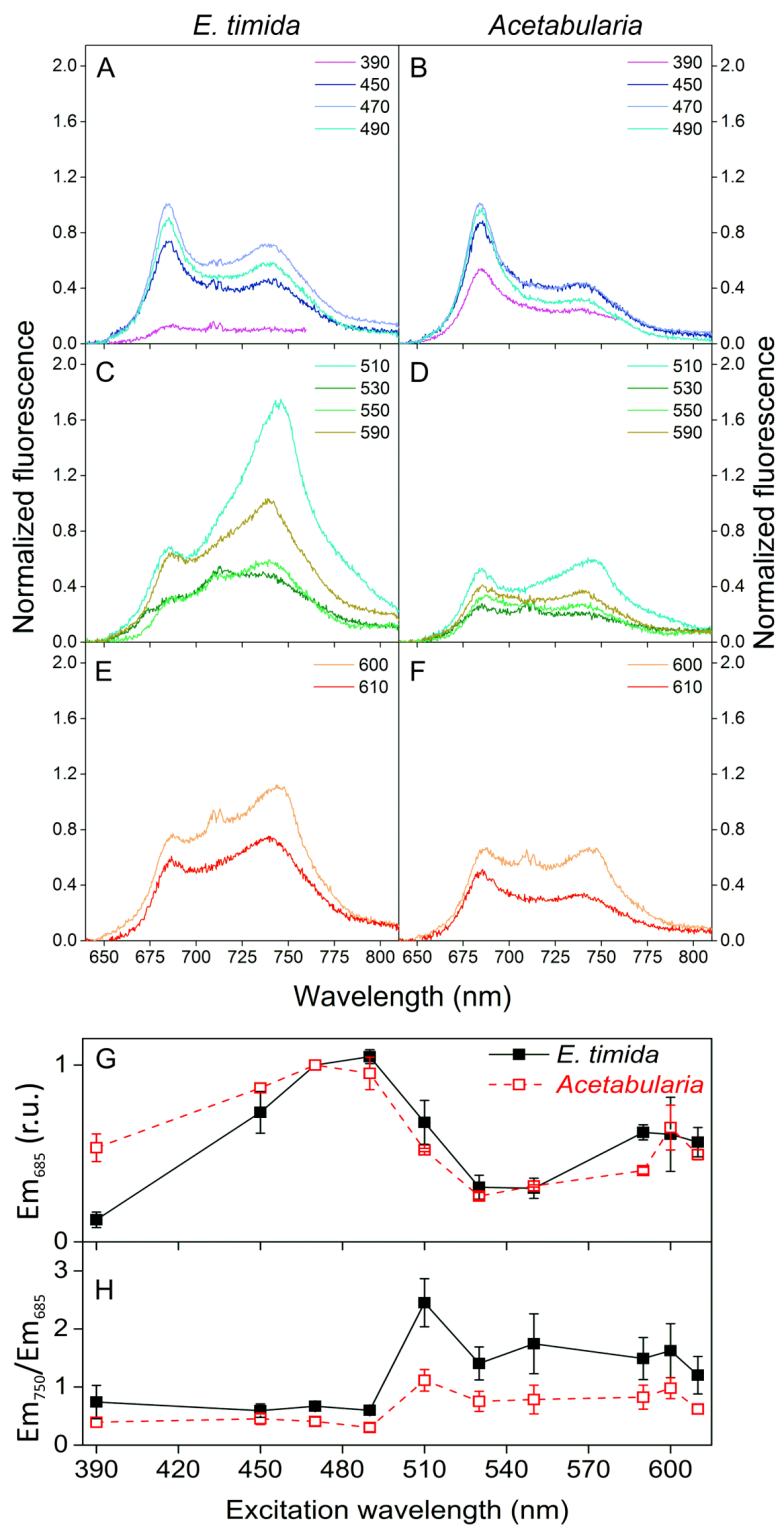
Room temperature Chl fluorescence emission from *E. timida* and *Acetabularia* samples excited with different wavelengths. **A**–**F** Fluorescence emission spectra from *E. timida* (left panels) and *Acetabularia* (right panels) after excitation with specific wavelengths of light, covering the UV and visible light regions. The excitation wavelengths are indicated in the legends. All fluorescence spectra were normalized to Chl fluorescence emission at 685 nm, excited by 470 nm light. **G** Excitation spectrum of fluorescence emission at 685 nm of *E. timida* (solid symbols) and *Acetabularia* (open symbols), normalized to fluorescence excited by 470 nm light. **H** The ratio of 750 nm to 685 nm fluorescence emission after excitation with different wavelengths. The data in panels **G** and **H** were derived from the measurements shown in panels **A**–**F**. The PFD of the 390 nm exciting light was 3 µmol m^−2^ s^−1^, whereas for all other wavelengths the PPFD was set to 4 µmol m^−2^ s^−1^. Each spectrum and data point represents an average of at least three biological replicates, and the error bars indicate SD. The double peak feature at around 710 nm, apparent when the fluorescence signal is low, is a reflected-light artifact

To elucidate the mechanisms protecting slug plastids against photoinhibition of PSII, we studied the penetration of different wavelengths to the slug tissue. For this, room temperature Chl fluorescence emission at different excitation wavelengths was measured (Fig. 3). Due to the nature of the samples, it was necessary to always perform two measurements (470 nm excitation as a control and another excitation wavelength) from each individual slug or algal mass. With the exception of the UV excitation, the first excitation wavelength was always 470 nm light to ensure that the samples were in correct position to emit a strong Chl fluorescence signal. For the next measurement from the same sample, the excitation light wavelength was changed to the desired one. This also enabled normalization of the fluorescence emission to the 470-nm-excited fluorescence at 685 nm, facilitating comparison between different samples.

In the tested UV to blue light excitation wavelengths (390–490 nm), the shapes of the fluorescence emission spectra of *E. timida* and *Acetabularia* showed peaks at the same positions, but the fluorescence emission peak at 750 nm was much more prominent in the slugs (Fig. 3A, B). UV radiation (390 nm) was strikingly less efficient in exciting Chl *a* fluorescence in the slugs than in the algae. In *E. timida* UV-excited fluorescence emission was very weak, whereas in *Acetabularia* the fluorescence yield under UV excitation was only slightly lower than under 470 nm excitation. This indicates that the harmful 390 nm UVA radiation is efficiently blocked from reaching the plastids inside the slugs.

### Fluorescence emission at 685 nm and light absorption per Chl are suppressed in the slugs

The fluorescence excitation spectra of *E. timida* and *Acetabularia* at 685 nm emission wavelength were similar, except for the weaker excitation efficiency of 390 nm in *E. timida* and somewhat weaker efficiency of 590 nm light in *Acetabularia* (Fig. 3G). This indicates that the light-harvesting properties of PSII are similar in both organisms. However, plotting the ratio of 750 nm to 685 nm fluorescence emission against the excitation wavelength revealed strong apparent suppression of 685 nm emission in the slugs (Fig. 3H). Such suppression (or apparent enrichment of 750 nm emission) effect can be the result of a higher local concentration of Chl (plastids) in *E. timida* in comparison to *Acetabularia*. High local concentration of Chl causes strong self-absorption of fluorescence emission at 685 nm, but not at 750 nm (Lichtenthaler et al. 1981; Weis 1985). Self-absorption also depends on the penetration depth of the excitation light: the deeper the excitation occurs, the higher is the probability of re-absorption of the fluorescence photon on its way out. This feature would also explain why 685 nm fluorescence is strongly suppressed especially in the green excitation wavelength region that is less efficiently absorbed by Chl than blue light (Fig. 3C, H).

**Fig. 4:**
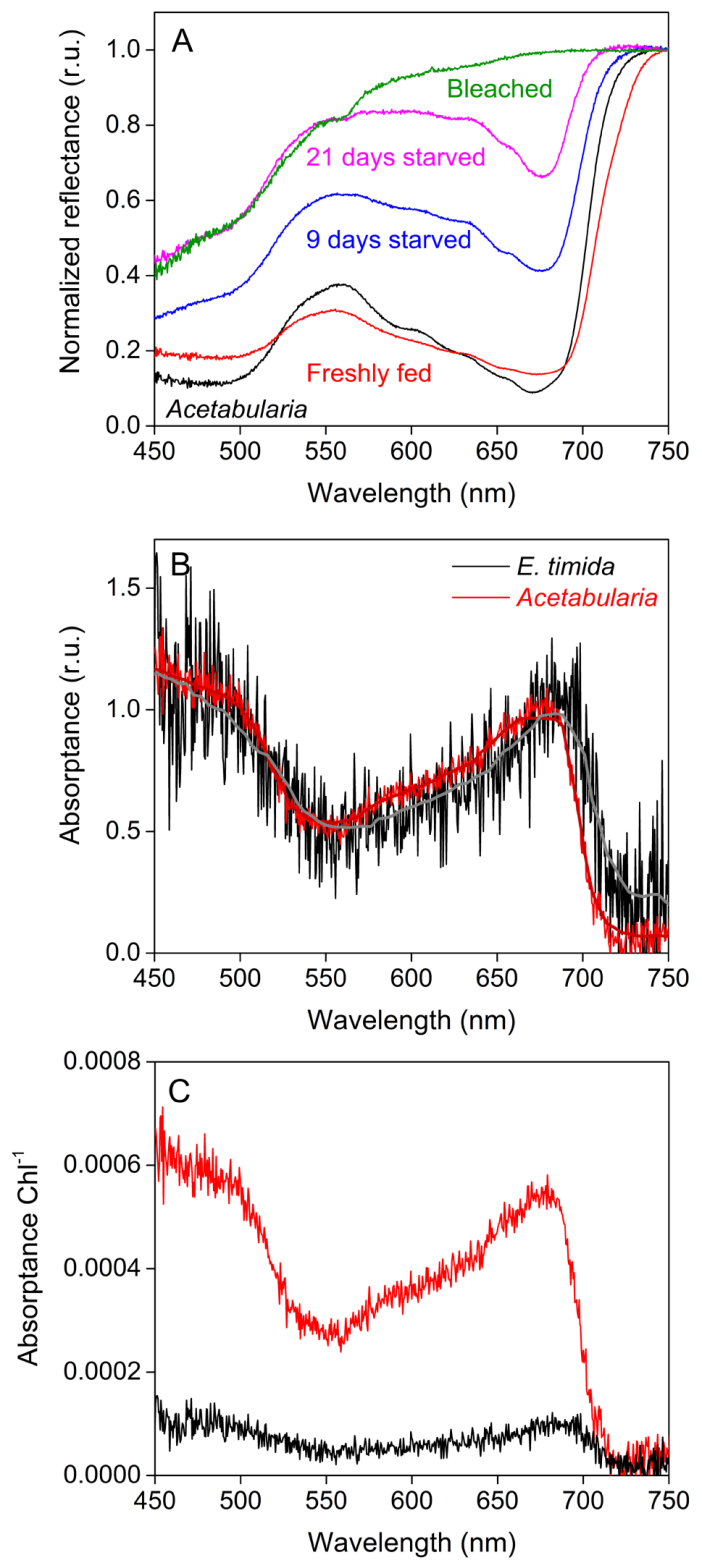
In vivo reflectance and absorptance spectra of *Acetabularia* and *E. timida.* **A** Spectral reflectance of *Acetabularia* (black) and *E. timida* individuals that were freshly fed (red), kept in starvation for 9 (blue) and 21 days (magenta), or until the slugs were almost completely bleached and devoid of plastids (green). All reflectance spectra were normalized to their respective reflectance at 750 nm. **B** Absorptance spectra, normalized to the red peaks at around 690 nm and 680 nm for *E. timida* (black) and *Acetabularia* (red)*,* respectively. The bold lines show a running median of the absorptance data. **C** The same spectra as in (**B**) normalized to the total Chl contents of the samples (87.83 µg Chl for *Acetabularia* and 210.52 µg Chl for *E. timida*). Each curve in (**A**) represents an average of at least three biological replicates. Each curve in (**B**) and (**C**) represents an average of three biological replicates for *Acetabularia*, whereas the *E. timida* spectrum represents an average of technical triplicates performed on a sample consisting of 30 slug individuals. Deviations have been omitted for clarity

The discussion above assumes that the absorption of light in the slugs in the 450 to 610 nm range is dominated by photosynthetic pigments. To test this, we measured both reflectance and absorptance spectra from intact slugs and pieces of *Acetabularia* (Fig. 4). The plastid density inside *E. timida* digestive tubules starts to decrease almost immediately after the slugs are deprived of their food (Laetz et al. 2017), which allows for a convenient way of inspecting the effect of plastid abundance on the spectral characteristics of the slugs. The reflectance spectra of freshly fed *E. timida* individuals and *Acetabularia* cells resembled each other, as both reflected far-red light (> 700 nm) and showed low reflectance in the red and blue regions due to absorption of light by Chl. Reflectance in the green region was higher than in red or blue but lower than in far-red, as expected for photosynthetic material (Virtanen et al. 2020). Intriguingly, the red edge of reflectance (the increase in reflectance at around 700 nm) in freshly fed slugs appeared to be shifted to longer wavelengths compared to *Acetabularia* (Fig. 4A). The optical properties of *E. timida* changed when the slugs were kept in starvation for 9 and 21 days, or until the slugs were nearly completely bleached, as shown by the reflectance spectra measured from these individuals (Fig. 4A). The difference in the position of the red edge of the reflection spectrum moved toward shorter wavelengths with the proceeding starvation. Interestingly, even the bleached slugs showed a decrease in reflectance with decreasing wavelength from red to blue light, and comparison of the reflection curves of 21 days starved and bleached slugs shows that in blue and green regions the effect of the remaining plastids on reflectance is negligible (Fig. 4A). Thus, the slug tissue absorbs some blue and green light but is transparent to red and far-red light.

The overall shapes of the absorptance spectra of *Acetabularia* and freshly fed *E. timida* were very similar, showing a distinctive red peak (approx. 650–690 nm), low absorptance in the green-yellow region (550–600 nm) and high absorptance in the blue region (450–500 nm) (Fig. 4B). This shape is to be expected for photosynthetic organisms that mainly rely on Chls *a* and *b* for light absorption, such as the green alga *Acetabularia*. However, in accordance with the red shift of the red edge of the reflectance spectra from freshly fed slugs, the red absorptance of the slugs peaked at around 690 nm, whereas in *Acetabularia* the red peak was clearly centered around 680 nm (Fig. 4B). Because of the considerably lower signal-to-noise ratio of the slugs compared to the algae (Fig. 4B), artefactual differences cannot be completely ruled out in the absorptance data. Even though the slug tissue itself was found to absorb blue and green light based on the reflectance data (Fig. 4A), this seems to be negligible in freshly fed slugs, where the shape of the absorptance spectrum is dominated by photosynthetic pigments, as in *Acetabularia* (Fig. 4B). When the absorptance data were normalized to the total Chl contents of the samples, it became evident that the slugs absorb a lot less light per Chl than the algae (Fig. 4C). Although the exact membrane systems surrounding the plastids in *E. timida* are still not resolved, plastids within this slug retain their spherical shape and thylakoid integrity (Wägele et al. 2011; Martin et al. 2013), suggesting that the lower absorption per Chl in *E. timida* than in *Acetabularia* is likely an indicator of tight, concentrated packing of the plastids in *E. timida* digestive tubules, not a result of changes in the plastid structure.

**Fig. 5:**
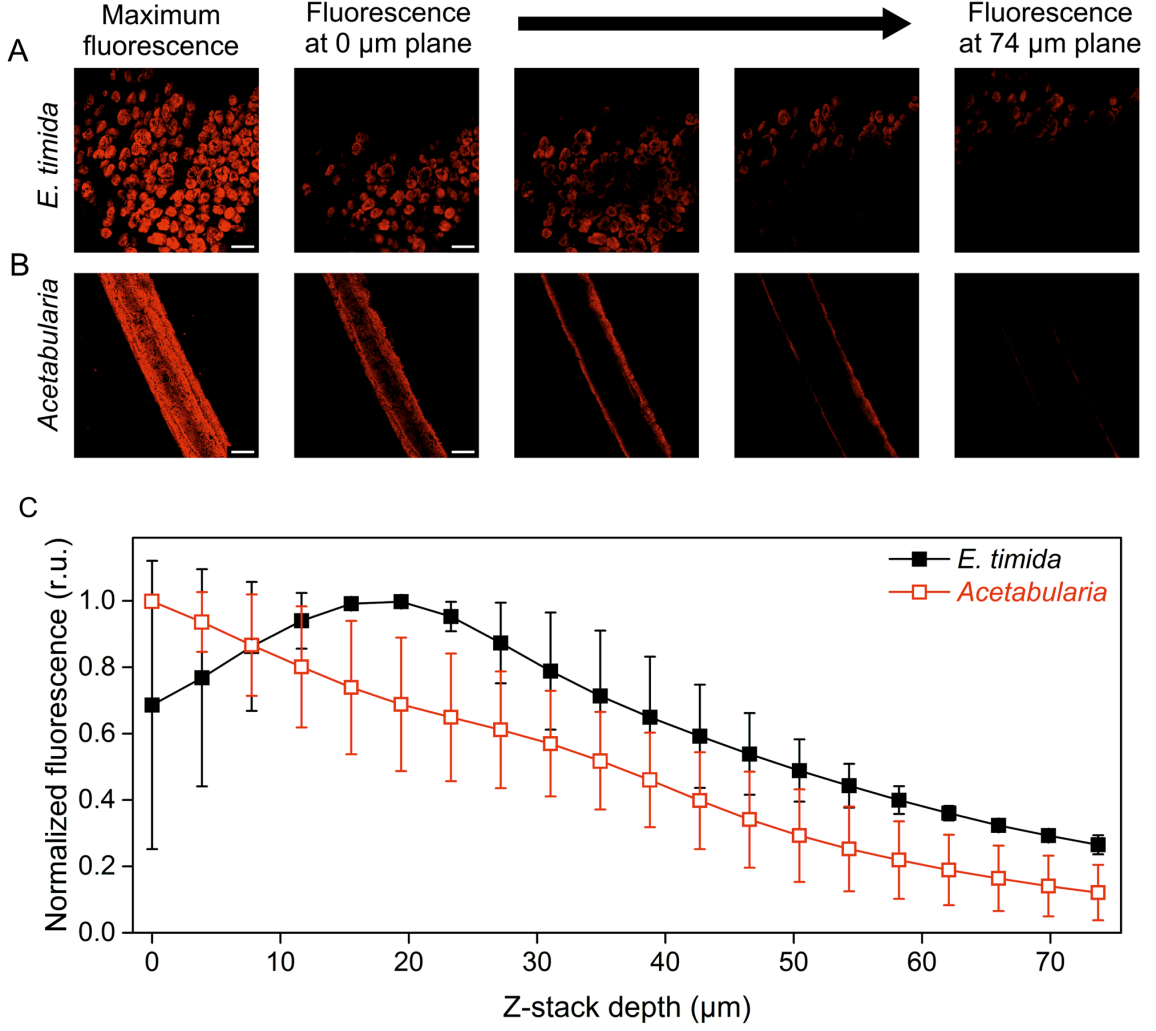
Confocal microscope imaging of Chl fluorescence at different depths inside *E. timida* tissues and *Acetabularia* cells. **A**, **B** Z-stack images from representative *E. timida* (**A**) and *Acetabularia* (**B**) samples. The first images on the left show the projected maximum Chl fluorescence stemming from each individual slice of the Z-stack. The subsequent four-image series (left to right) show Chl fluorescence of individual layers of the Z-stack from the beginning of the stack (0 µm plane) to the end (74 µm plane). The images in between are intermediates at different planes. The scale bars equal 1000 µm. **C** Average Chl fluorescence emission at slices of the Z-stacks at different depths inside the *E. timida* (solid symbols) and *Acetabularia* (open symbols) samples, normalized to their respective maxima. Each data point in (**C**) represents an average of two biological replicates, and the error bars show SD

We also investigated the distribution of plastids inside freshly fed *E. timida* individuals and *Acetabularia* cells using confocal microscopy (Fig. 5). Even though the actual plastid concentration could not be calculated from the micrographs, the images suggest that in the slugs the plastids are arranged in multiple layers within the body, whereas in the algae most of the plastids reside within a narrow layer within the algal cell. Inspecting Chl fluorescence of individual slices of the Z-stack (20 slices spanning 74 µm at intervals of 3.8 µm) revealed that the fluorescence signal in the slugs stayed strong in a wide depth range, whereas the fluorescence signal in *Acetabularia* decreased almost linearly throughout the Z-stack (Fig. 5C).

### Starvation makes E. timida susceptible to visible light-induced photoinhibition but has a smaller effect on UVA-induced photoinhibition

**Fig. 6:**
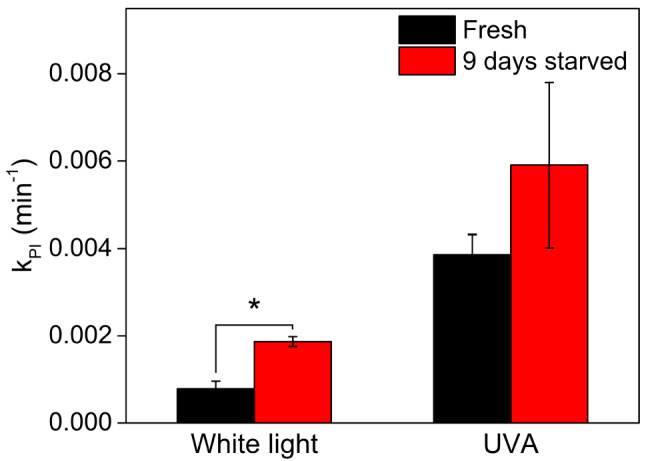
The effect of starvation on susceptibility to photoinhibition in *E. timida*. Rate constant of photoinhibition (k_PI_) induced by white light or UVA, as indicated, of freshly fed *E. timida* slugs (black) and after 9 days in starvation (red). All k_PI_ values were normalized to P(P)FD 300 µmol m^−2^ s^−1^; the actual PPFD of the white light was 900 µmol m^−2^ s^−1^, and the PFD of the UVA radiation treatment was 33 µmol m^−2^ s^−1^. A significant difference between the treatments is indicated by an asterisk (*P < 0.01, Welch’s t-test). Each bar represents an average of four biological replicates and the error bars indicate SD

As the changes in the reflectance spectra of *E. timida* kept in starvation for 9 and 21 days indicated a decrease in the plastid content of the slugs (Fig. 4A), we illuminated these starved slugs in the presence of lincomycin to test if a high plastid concentration protects against photoinhibition. The results show that slugs that had been kept in starvation for 9 days were significantly (*P* < 0.01, *n* = 4, Welch’s t-test) more susceptible to photoinhibition in visible light (PPFD 900 µmol m^−2^ s^−1^) than freshly fed slugs (Fig. 6), and after 21 days the rate constant of photoinhibition (k_PI_ normalized to 300 µmol m^−2^ s^−1^ = 0.01 min^−1^, SD < 0.01, *n* = 7) was approximately seven times as high as that of freshly fed slugs. The 21-day data may not be equally significant as the 9-day data because the *F*_V_/*F*_M_ of the slugs had already started to decrease during the 21 days of starvation (*F*_V_/*F*_M_ = 0.50, SD ± 0.06, *n* = 7), and their susceptibility to photoinhibition might be affected by a multitude of factors. When 9 days starved slugs were subjected to UVA (365 nm, PFD 33 µmol m^−2^ s^−1^), k_PI_ was not significantly higher than in freshly fed slugs, although clearly increasing (Fig. 6). The difference between UVA and visible light suggests that a major factor in protecting the plastids against visible light inside the slugs is the high initial plastid concentration in their tissues, whereas the UV protection is caused by the absorption of UV radiation by the slug tissue or mucus. However, as the UVA photoinhibition did also increase in the starved slugs, it is likely that both UV screening and plastid packing contribute to resilience against UV photoinhibition.

## Discussion

### Generalities of photoinhibition hold true for E. timida

Three aspects of photoinhibition are nearly ubiquitous among photosynthetic organisms: (I) photoinhibition in the presence of plastid specific translation inhibitors, such as lincomycin, proceeds according to first-order reaction kinetics, (II) k_PI_ is directly proportional to PPFD, and (III) UV radiation causes more damage to PSII than visible light (Tyystjärvi 2013). All of these “rules” also govern the damage to PSII in the photosynthetic sea slug *E. timida* and its prey, the green alga *Acetabularia* (Figs. 1 and 2). This indicates that the slugs do not alter the fundamental energetic processes of the plastids in a way that would cause deviations to these core properties.

Some photosynthetic slugs, like *E. viridis*, have been shown to either curl up or move to a shadier area when exposed to strong light (Cruz et al. 2013; Cartaxana et al. 2018), and it was recently shown that also *E. timida* individuals close their parapodia, the wing-like appendices on their sides, in response to increasing light intensity (Cartaxana et al. 2019). The authors suggested that this is a photoprotective response. Even though we did not measure the exposed dorsal area of the slugs, we did witness similar behavior during the photoinhibition experiments, especially at the light treatments with PPFD > 1000 µmol m^−2^ s^−1^. However, k_PI_ was directly proportional to PPFD (Fig. 1), although deviation from linearity would be expected if the shift from open to increasingly closed parapodia was a major photoprotective measure against photoinhibition of PSII. It should be noted that even the lowest PPFD in our light response curve was 300 µmol m^−2^ s^−1^, which exceeds the range where Cartaxana et al. (2019) reported a major shift from open (PPFD < 200 µmol m^−2^ s^−1^) to closed parapodia (PPFD > 200 µmol m^−2^ s^−1^) in *E. timida*, possibly suggesting that the resolution of our light response curve may not be sufficient to catch the protective effect of the animal behavior against photoinhibition. Considering the very slow photoinhibition that the dimmest lights used in our study caused in the slugs (Fig. 1A), it might be very difficult to discern the protective effects that the closure of the parapodia possibly have with the methodology used in the current study.

### *Elysia timida* body offers efficient UV sunscreen for its plastids

A large portion of the studied green macroalgae species efficiently repair UV-induced damage rather than screen UV radiation (Pescheck et al. 2010, 2014). Porst et al. (1996) showed that *Acetabularia mediterranea* is photoinhibited more by UVB than UVA, and this species recovers from photoinhibition caused by strong natural sunlight almost completely within two hours in the shade. The samples used by Porst et al. (1996) were grown naturally in the sea, and therefore likely to have been exposed to at least some UV radiation during their lifetime, which might indicate that also *A. mediterranea* relies on efficient repair instead of UV screening. Likewise, our data show that UVB (and UVC) cause more photoinhibition than UVA in *Acetabularia* (Fig. 2).

Sea slugs capable of long-term retention of plastids, including *E. timida*, have genes for a fatty acid synthase-like polyketide synthase (FAS-like PKS), suggesting that they use methylmalonyl-CoA as a substrate to produce polypropionates that can be converted to specific complex polyketides only found in these photosynthetic sea slugs (Torres et al. 2020). The presence of complex polyketides in *E. timida* has also been confirmed, and the compounds identified in it include *ent*-9,10-deoxytridachione, tridachione, photodeoxytridachione and 15-norphotodeoxytridachione (Gavagnin et al. 1994; Torres et al. 2020). These compounds have been suggested to function as UV sunscreens in the mucus excreted by the slugs (Ireland and Scheuer 1979), and they are related to the plastids, as the mucus contains a large fraction of the radiolabeled carbon originating from carbon fixation by the slug plastids (Trench et al. 1972). Accordingly, our results show that the slug tissue efficiently blocks UV radiation from reaching the plastids (Fig. 3) thereby protecting the plastids from UV-induced photoinhibition (Fig. 2). The protection appears to function even in starved slug individuals to some extent, suggesting that this protection mechanism contributes to resilience against photoinhibition in conjunction with the mechanism that protects in visible light (Fig. 6). UV radiation, mostly UVA and UVB, is a common stressor for both photosynthetic and non-photosynthetic organisms inhabiting shallow intertidal waters (Häder et al. 2007) and is expected to also affect photosynthetic sea slugs. Screening of UV radiation may have an effect on plastid longevity in *E. timida* in their natural habitat, as sunlight has a considerable UVA contribution and UVA is efficient in causing photoinhibition (Fig. 2). Decreased UV photoinhibition could contribute to allowing the slugs to maintain their plastids longer in times of food shortage. However, our data do not allow us to identify the screening molecules, and a comparison of plastid longevity in natural sunlight with and without UV radiation is needed to fully determine if the UV screening has a pronounced physiological role.

If *Acetabularia* itself has evolved to deal with UV radiation by efficient repair, then plastids in *E. timida* would benefit from both efficient repair machinery of the alga (at least to the extent that is possible without the algal nucleus) and UV screening of the slug. While the genetic autonomy and efficient repair machinery of *Vaucheria litorea* plastids are likely a major factor in maintaining the plastids functional in the sea slug *E. chlorotica* (Green et al. 2000; Havurinne et al. 2021), there are contrasting reports on the capability of plastids inside *E. timida* to recover from photoinhibition. Christa et al. (2018) incubated *E. timida* (and also *E. viridis*) slugs in lincomycin for 2 h in the dark prior to a 1 h photoinhibition treatment (red light, PPFD 1300 µmol m^−2^ s^−1^) followed by a 30 min recovery period in the dark and found no difference in the repair of photodamage in *E. timida* in the presence or absence of lincomycin. Our previous results suggest otherwise, but it should be noted that the experimental design in our study was different from the one in Christa et al. 2018. In our study the slugs were incubated overnight in lincomycin in the dark and then exposed to high light (white light, PPFD 2000 µmol m^−2^ s^−1^, measured with a planar sensor) for 40 min. The slugs were allowed to recover overnight in low light in their growth conditions, where they were subjected to approximately 10 h of light (PPFD < 20 µmol m^−2^ s^−1^ during the day) and 12 h of darkness (during the night), and in our experiment lincomycin strongly inhibited the recovery (Havurinne et al. 2021). We do agree with the statement made in Christa et al. (2018) that the plastids inside *E. timida* do not recover as efficiently as they do inside *Acetabularia* (Fig. S1 in Online Resource), but the data in Havurinne et al. (2021) show that the inherent repair machinery of the plastids does likely play a role in maintaining the plastids functional also in *E. timida*, at least in freshly fed slugs.

### Tight packing of plastids within E. timida protects from photoinhibition

*Acetabularia* cells appear transparently green, whereas the areas with plastids in the slugs are bright green. The spectral characteristics of freshly fed *E. timida* and *Acetabularia* corroborate these ocular observations and show that the plastids of *E. timida* are tightly packed, in comparison to the plastids within their original host. Firstly, *E. timida* exhibited a strong suppression of 685 nm fluorescence due to self-absorption compared to *Acetabularia* in the tested excitation wavelength range (Fig. 3). Reflectance and absorptance spectra provide a second piece of evidence for tight packing of plastids within the slugs, as the red edge of reflectance and the red absorption peak of Chl *a* in freshly fed *E. timida* are shifted in a manner that suggests that the slugs absorb longer wavelengths of red and far-red light than *Acetabularia* (Fig. 4A, B). The same phenomenon can be seen in senescing birch leaves, as green leaves absorb light at longer wavelengths than senescing ones (Mattila et al. 2021). Furthermore, the red edge of the reflectance spectrum of *E. timida* moves toward shorter wavelengths when the slugs lose plastids during starvation (Fig. 4A). These data show that the position of the red edge of reflectance, and consequently also that of the red peak of absorptance, depend on the amount of green plastids per slug. Similar changes in reflectance spectra during starvation have been noted also in *E. viridis*, suggesting that the phenomenon and associated photoprotection by the outer plastids might be relevant for other photosynthetic slugs as well, not just *E. timida* (Serôdio et al. 2010). Tight packing of slug plastids also explains why the slugs absorb much less light than the algae when the absorptance is normalized to the Chl content of the samples (Fig. 4C). It should be noted, however, that 30 slugs had to be packed in a test-tube to get a single absorptance reading, and therefore the packing of slugs may have further lowered the absorptance. Results of confocal microscopy further confirm that plastids inside *E. timida* are spread to a wider depth range than plastids in *Acetabularia* (Fig. 5).

Tight packing of plastids inside the slug tissue can explain why the slug plastids appear to be less prone to photoinhibition than the same plastids in *Acetabularia* (Fig. 1; Christa et al. 2018). The mechanism is simple: the outermost plastids of a tight stack prevent light or UV radiation from reaching the lower ones. The rate of photoinhibition depends on the intrinsic susceptibility of each PSII and on the photon flux reaching each PSII. The latter depends on the optical thickness of the sample and on Chl distribution within it (Pätsikkä et al. 2002; Serôdio et al. 2014; Serôdio and Campbell 2021). In the case of *E. timida*, protection by high optical thickness can be described as outer plastids protecting the inner ones.

The *F*_V_/*F*_M_ parameter is often assumed to reflect the state of the outermost layers of the plastids of the sample. In this line, protection found by *F*_V_/*F*_M_ measurements might suggest that the same plastids do not serve as protecting agents at all times, but their exposure levels become mixed when the slug moves. However, recent analysis shows that *F*_V_/*F*_M_ measurements probe photoinhibition deeper than expected, thereby allowing quantification of a depth-integrated rate of photoinhibition rather than photoinhibition of the outermost layers only (Serôdio and Campbell 2021). For these reasons, potential effects of kleptoplasty on the inherent susceptibility of PSII to photoinhibition remain to be elucidated, but the plastids are nevertheless likely protected from photoinhibition by the optical thickness of the plastid layer. The finding that the susceptibility of algal plastids to visible light photoinhibition increases when plastids are lost during starvation (Fig. 6) confirms that the packing of plastids protects their PSII against photoinhibition. Our hypothesis also predicts that the plastids occupying the outermost digestive tubule cells of the slugs should experience more photoinhibition than the inner ones, and therefore the outer ones are likely degraded faster than the inner ones. This should be tested in future studies.

Tight packing of plastids as a major mechanism of protection against photoinhibition of PSII does not exclude possible protection by other mechanisms. Plastids inside *E. timida* maintain physiological photoprotection mechanisms, such as the xanthophyll cycle, a major constituent of NPQ (Christa et al. 2018; Cartaxana et al. 2019). However, the effect of NPQ on photoinhibition of PSII is usually small (Tyystjärvi 2013), and other photoprotective mechanisms found in slug plastids (Havurinne and Tyystjärvi 2020) would only marginally protect PSII. Our data suggest that, if given the chance, *E. timida* slugs fill their thick bodies up with plastids, which protects the plastids from photoinhibition of PSII. Slow photoinhibition improves the longevity of the plastids inside photosynthetic sea slugs and alleviates the need for an efficient PSII repair cycle that may still contribute to the functionality of the plastids, at least in the beginning of starvation.

Photosynthetic sea slugs can move away from excessive irradiation if the need arises. Nevertheless, periods of strong light are inevitable in the shallow waters that slugs like *E. timida* inhabit. Our results demonstrate that the slugs protect their plastids by screening highly damaging UV radiation and by packing their plastids tightly all over their bodies, allowing the outer layers to take the brunt of the damage.

## Supplementary Information

Below is the link to the electronic supplementary material.

Supplementary file1 (PDF 671 kb)

## Data Availability

Original data are available in Mendeley Data at https://doi.org/10.17632/8mwgdskw2x.1.

## Code availability

No custom code was used.
