# Supplementary material for: Ultraviolet screening by slug tissue and tight packing of plastids protect photosynthetic sea slugs from photoinhibition
Source: Photosynth Res. 2021 Nov 26;152(3):373–87. doi: 10.1007/s11120-021-00883-7 (PMC9458594; doi:10.1007/s11120-021-00883-7)
Supplement: Supplementary file 1 — Supplementary file1 (PDF 671 kb) [file 11120_2021_883_MOESM1_ESM.pdf]

Supplementary information

Title: Ultraviolet screening by slug tissue and tight packing of plastids protect photosynthetic sea slugs from photoinhibition

Journal: Photosynthesis Research

Authors: Vesa Havurinne, Riina Aitokari, Heta Mattila, Ville Käpylä, Esa Tyystjärvi*

Corresponding author: Esa Tyystjärvi; University of Turku, Department of Life Technologies/Molecular Plant Biology, Finland; email: esatyy@utu.fi

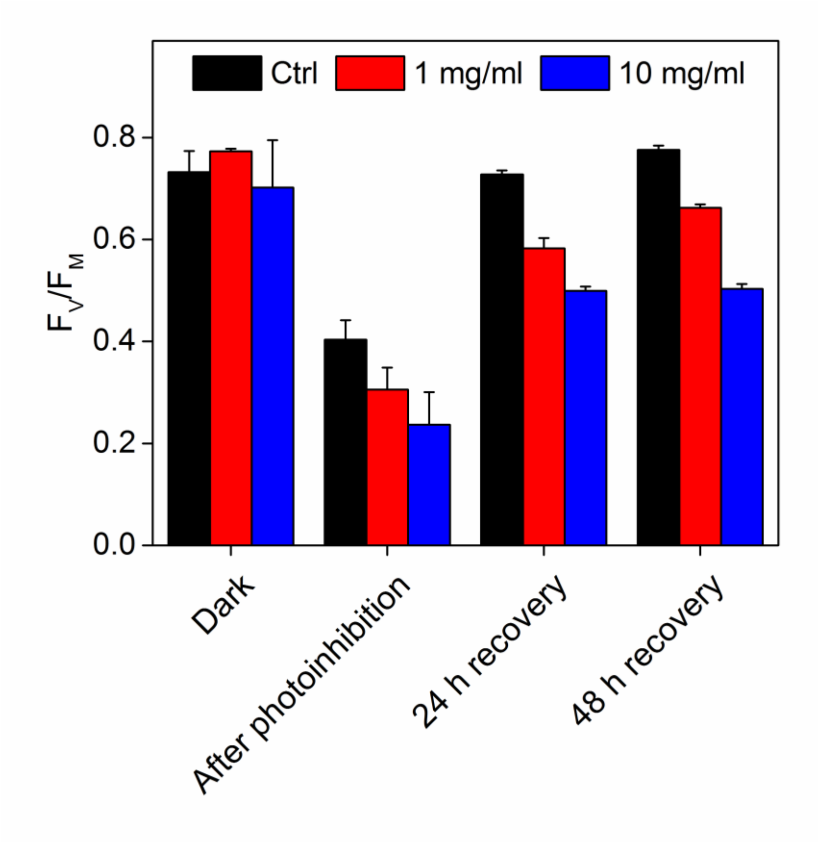

Supporting information fig. S1 The effect of different concentrations of lincomycin on recovery of PSII activity in *Acetabularia.* All samples were kept in the dark overnight in the absence (control, black bars) or presence of 1 mg/ml (red bars) or 10 mg/ml (blue bars) of lincomycin in f/2 culture medium prior to exposing them to a 60 min high light treatment (PPFD 6000 µmol $m^{-2}s^{-1}$). The same samples were subsequently incubated in the presence of lincomycin in the growth conditions under low light (PPFD ~10 µmol $m^{-2}s^{-1}$) to measure recovery from photoinhibition. The fluorescence parameter F_V/F_M

was used as a proxy of PSII activity, and it was measured from the samples after a minimum of 20 min dark period. Each bar represents an average of three biological replicates and the error bars show SD

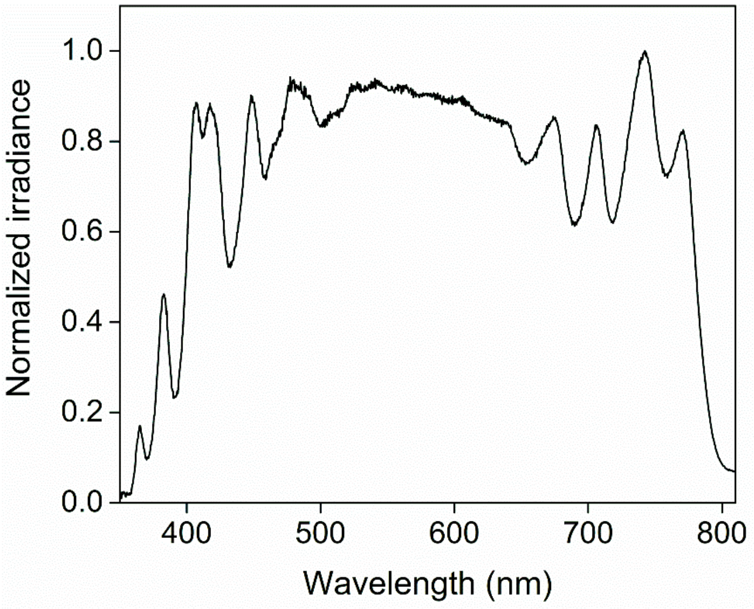

Supporting information fig. S2 Normalized irradiance spectrum of the Artificial Sunlight Module (SLHolland) that was used as a light source for the white light photoinhibition treatments
